# Amino acid transporter SLC7A5 regulates cell proliferation and secretary cell differentiation and distribution in the mouse intestine

**DOI:** 10.7150/ijbs.94297

**Published:** 2024-03-25

**Authors:** Lingyu Bao, Liezhen Fu, Yijun Su, Zuojia Chen, Zhaoyi Peng, Lulu Sun, Frank J. Gonzalez, Chuan Wu, Hongen Zhang, Bingyin Shi, Yun-Bo Shi

**Affiliations:** 1 Section on Molecular Morphogenesis, Eunice Kennedy Shriver National Institute of Child Health and Human Development, National Institutes of Health, Bethesda, Maryland, MD, USA.; 2 Department of Endocrinology, The First Affiliated Hospital of Xi'an Jiaotong University School of Medicine. No.277, Yanta West Road, Xi'an, Shaanxi, 710061, P.R. China.; 3 Laboratory of High Resolution Optical Imaging and Advanced Imaging and Microscopy Resource, National Institute of Biomedical Imaging and Bioengineering, National Institutes of Health, Bethesda, MD, 20892, USA.; 4 Experimental Immunology Branch, National Cancer Institute, National Institutes of Health, Bethesda, MD 20892, USA.; 5 Laboratory of Metabolism, Center for Cancer Research, National Cancer Institute, National Institutes of Health, Bethesda, MD 20892, USA.; 6 Bioinformatics and Scientific Programming Core, Eunice Kennedy Shriver National Institute of Child Health and Human Development, National Institutes of Health, Bethesda, Maryland, USA.; 7 Janelia Research Campus, Howard Hughes Medical Institute (HHMI), Ashburn, VA, USA.

**Keywords:** amino acid transporter, intestine, organ homeostasis, mTORC1 signaling pathway, stem cell

## Abstract

The intestine is critical for not only processing nutrients but also protecting the organism from the environment. These functions are mainly carried out by the epithelium, which is constantly being self-renewed. Many genes and pathways can influence intestinal epithelial cell proliferation. Among them is mTORC1, whose activation increases cell proliferation. Here, we report the first intestinal epithelial cell (IEC)-specific knockout (^ΔIEC^) of an amino acid transporter capable of activating mTORC1. We show that the transporter, SLC7A5, is highly expressed in mouse intestinal crypt and *Slc7a5*^ΔIEC^ reduces mTORC1 signaling. Surprisingly, adult *Slc7a5*^ΔIEC^ intestinal crypts have increased cell proliferation but reduced mature Paneth cells. Goblet cells, the other major secretory cell type in the small intestine, are increased in the crypts but reduced in the villi. Analyses with scRNA-seq and electron microscopy have revealed dedifferentiation of Paneth cells in *Slc7a5*^ΔIEC^ mice, leading to markedly reduced secretory granules with little effect on Paneth cell number. Thus, SLC7A5 likely regulates secretory cell differentiation to affect stem cell niche and indirectly regulate cell proliferation.

## Introduction

The intestine is the organ responsible for the digestion and absorption of nutrients and water, and also forms a barrier against environmental harmful materials/organisms. It is under a constant barrage of mechanical, chemical, and pathogen-driven attacks from food digestion and the trillions of microorganisms that colonize the intestine 1, 2. It is thus critical to maintain intestinal integrity and function. Intestinal homeostasis relies on a perfect balance of the interactions among different cell types within intestinal epithelium, the underlying non-epithelial tissues, the microbiota, and immune system 2-5. Intestinal epithelial cells are crucial for intestinal homeostasis and are constantly renewed, every 3-5 days in the case of mice, driven by adult stem cells 6, 7. Two types of epithelial cells, goblet cells and Paneth cells, function to maintain intestinal integrity. Goblet cells secret mucus that coats the epithelium to block the access of the microbiota to mucosa and to allow the accumulation of high concentrations of anti-microbial peptides (AMPs). Reduced goblet cell numbers and mucus secretion are the hall marks of human inflammatory bowel disease (IBD) 8. Paneth cells secrete AMPs, including lysozyme and defensins, that protect the epithelium from pathogens. Recent studies have shown that impaired AMP secretion may contribute to IBD susceptibility 9, 10.

SLC7A5, also known as LAT1, forms a heterodimeric amino acid transporter by interacting with glycoprotein CD98 (SLC3A2) involving a conserved disulfide bond 11. The heterodimer transports large neutral amino acids and is crucial for immune system, placenta, blood brain barrier, and protecting the body against pathogens 12-14 and disruption of the transporter function by disrupting the extracellular domain of CD98 leads to embryonic lethality in mouse 15. SLC7A5-mediated mTORC1 activation is well known to be important for immune system, especially T cell activation 16. SLC7A5 is broadly expressed in many organs, including the intestine 17. However, if and how SLC7A5-mediated mTORC1 activation plays a role in intestinal homeostasis and function remain unknown. While there have been studies on mTORC1 signaling in intestine, they have been mainly focused on energy utilization with not very consistent findings. For example, it was shown that calorie restriction leads to inhibition of mTORC1 pathway specifically in Paneth cells, which in turn increases crypt cell proliferation and LGR5+ stem cell numbers through activating SIRT1 18-20. On the other hand, a different study showed that calorie restriction reduced cell proliferation, increased reserve stem cells (BMI1+ stem cells) other than LGR5+ stem cells in the intestine 21. Thus, further analyses are needed to understand the effects and regulation of mTORC1 signaling in intestinal homeostasis.

Here, we made use of a floxed *Slc7a5* allele (*Slc7a5*^fl/fl^) 17 to knock out *Slc7a5* specifically in the intestinal epithelium by crossing the *Slc7a5*^fl/fl^ line with a villin-cre line, where the Cre recombinase is under the control of intestinal epithelial cell (IEC)-specific villin promoter, to generate *Slc7a5*^ΔIEC^ mice. This allowed us to investigate if epithelial expression of this amino acid transporter affects mTORC1 function and/or intestinal development and/or homeostasis. Our study showed that loss of SLC7A5 in intestinal epithelium led to reduced mTORC1 signaling and a dramatic decrease in the number of mature secretory cells, including Paneth cells and goblet cells in the small intestine, although the goblet cells was increased in the crypts. In addition, as mTORC1 is a well-known, important pathway for cell proliferation, we examined cell proliferation in the intestine and found that surprisingly, loss of SLC7A5 in intestinal epithelium increased cell proliferation in the crypts. Furthermore, we observed increased cell death in the epithelium and faster epithelial cell turnover. On the other hand, the number of crypt base columnar (CBC) stem cells, such as LGR5 positive and OLFM4 positive stem cells, were not changed. Our findings thus revealed that knocking out SLC7A5 in intestinal epithelium affected intestinal defense function via reduced mTORC1 signaling in secretory cell lineage and further suggest a model where a complex and delicate intestinal regulatory system helps to maintain physiological or pathophysiological status of the intestine.

## Results

### Intestinal epithelium-specific knockout of SLC7A5 leads to longer crypts in mice

**Figure 1:**
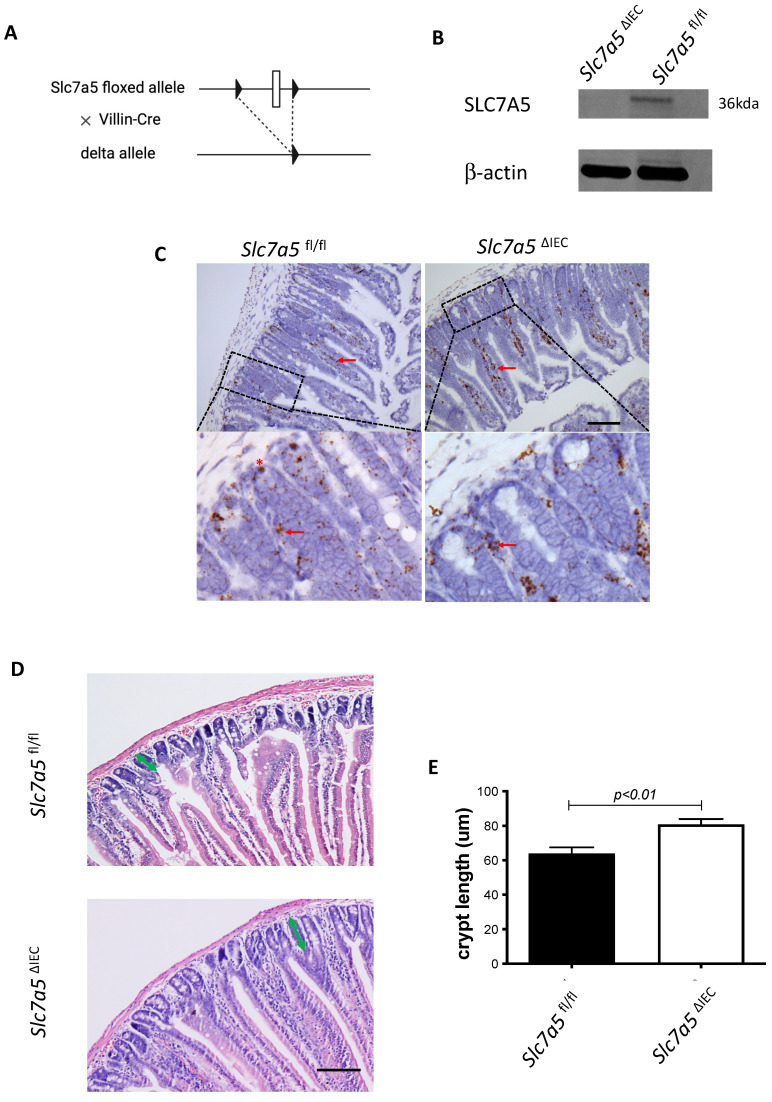
** Intestinal epithelial cell specific knockout of SLC7A5 leads to more elongated crypts in the small intestine. A**. Schematic diagram of the *Slc7a5*-floxed locus in *Slc7a5*^fl/fl^ mice 17 used to generate *Slc7a5*^ΔIEC^ mice. Two loxP sites (black triangle) flanked *Slc7a5* exon 1. When *Slc7a5*^fl/fl^ mice are crossed with mice containing Cre under the control of the epithelial specific villin promoter (Villin-Cre), the exon flanked by the two loxP sites is removed to generate the deleted allele. **B**. *Slc7a5*^ΔIEC^ mice have little or no detectable SLC7A5 protein in the intestinal epithelium. Intestinal epithelium was isolated from *Slc7a5*^ΔIEC^ and *Slc7a5*^fl/fl^ mice and its proteins were subjected to western blot analysis for *SLC7A5* expression, with β-actin as a loading control. **C**. SLC7A5 is expressed in both epithelium (red asterisk) and submucosal area (red arrows) and *Slc7a5*^ΔIEC^ eliminates its epithelial expression. Intestinal cross-sections were subjected to immunohistochemical analysis for SLC7A5 expression. Note that *Slc7a5*^ΔIEC^ reduced/abolished SLC7A5 in the crypt epithelium but not in the submucosal area, e.g., immune cells (red arrows). Scale bars, 100μm. **D-E**. *Slc7a5*^ΔIEC^ mice have longer crypts in the small intestine. H&E staining of intestine from *Slc7a5*^fl/fl^ and *Slc7a5*^ΔIEC^ mice **D**. showing longer crypts (green double headed arrows). The crypt lengths were measured (n=5) and shown as mean ± SD (E).

Whole body knockout of SLC7A5 causes embryonic lethality in mice 22. To study the role of SLC7A5 in the intestine, we crossed villin-Cre mice with *Slc7a5*^fl/fl^ mice to delete the floxed exon in *Slc7a5* gene specifically in intestinal epithelium (Fig. 1A). Homozygous *Slc7a5*^ΔIEC^ mice were born at the Mendelian ratios and developed normally. Western blot analysis of isolated small intestinal crypts showed that SLC7A5 protein was expressed in the epithelium of wild-type *Slc7a5*^fl/fl^ mice but absent in *Slc7a5*^ΔIEC^ mice (Fig. 1B), demonstrating the knockout efficiency. Furthermore, immunohistochemical analysis showed that in adult intestine, SLC7A5 was found in both epithelium and submucosal area of wild-type mice but absent in the epithelium of the *Slc7a5*^ΔIEC^ mice (Fig. 1C). The *Slc7a5*^ΔIEC^ mice were phenotypically normal externally throughout development and adulthood (not shown). In addition, isolated intestine also had normal gross morphology (not shown). On the other hand, H&E-stained histological sections of the small intestine showed that the *Slc7a5*^ΔIEC^ intestine had longer crypts (Fig. 1D).

### *Slc7a5*^ΔIEC^ mice have increased cell proliferation in the crypt and accelerated epithelial cell turn-over with compensating increases in cell death in the epithelium

**Figure 2:**
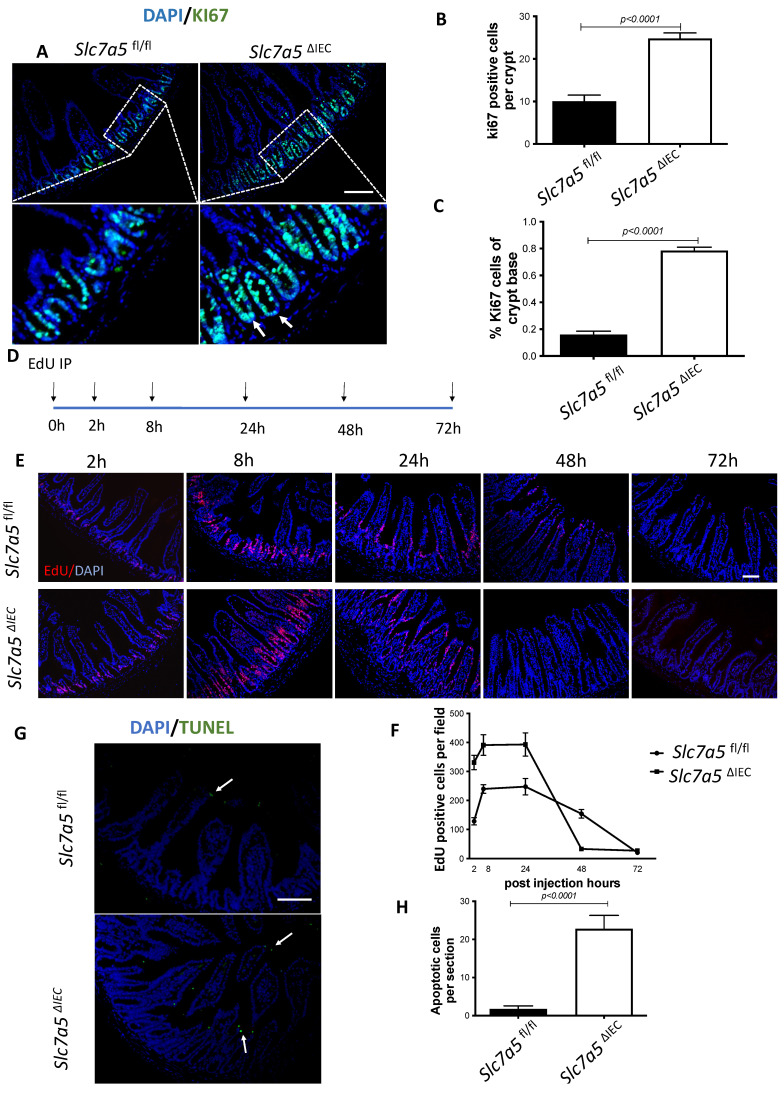
***Slc7a5*^ΔIEC^ mice have faster epithelial cell turnover, with increased cell proliferation and epithelial cell death. A-C**. Cross-sections of the small intestine were labeled with DAPI (blue) for DNA and anti-Ki67 antibody (green) for proliferating cells (**A**), showing more cell proliferation in the crypt of *Slc7a5*^ΔIEC^ mice. The proliferating cells in the crypt (**B**) or at crypt base (**C**) were quantified, revealing that *Slc7a5*^ΔIEC^ mice had increased Ki67 staining in both transit amplifying (TA) cell zone and crypt base, where crypt base stem cells and Paneth cells are located, respectively. Scale bars, 100μm. **D**. Schematic diagram of EdU time course experiment. EdU was injected at 0 hr and the intestine was isolated at indicated time points (arrows) for analysis **E**. EdU staining of small intestine from *Slc7a5*^fl/fl^ and *Slc7a5*^ΔIEC^ mice at 2 h, 8 h, 24 h, 48 h and 72 h after Edu injection. Note that compared to *Slc7a5*^fl/fl^ mice, *Slc7a5*^ΔIEC^ mice had increased EdU-labeling in the crypts at 2-8 h, indicative of increased cell proliferation. The EdU-labeling reached plateau after 8 h, suggesting that the unincorporated EdU had been metabolized. The EdU-labeled cells in *Slc7a5*^ΔIEC^ intestine, however, had faster migration, with many of them differentiated and migrated into the villi by 8 h. Scale bars, 100μm. **F**. Quantification of EdU-labeled cells from intestinal cross-sections as shown in E. Note that *Slc7a5*^ΔIEC^ mice had increased cell proliferation (2-8 h) and faster turnover rate, with essentially all EdU-labeled cells eliminated by 48 h. G-H. TUNEL assay showing increased apoptotic cells in small intestine of *Slc7a5*^ΔIEC^ mice compared to *Slc7a5*^fl/fl^ mice (**G**), with the quantification shown mean ± SD (**H**). Scale bars, 100μm.

Generally, the upper two thirds of the crypt is referred to as the transit amplifying cell zone, where the cells, called transit amplifying cells, proliferate rapidly and therefore can be visualized with Ki67 labeling (Fig. 2A). In *Slc7a5*^ΔIEC^ mice, the Ki67 positive cells increased by about two-fold compared to that in *Slc7a5*^fl/fl^ mice (Fig. 2B). In addition, while rare, Ki67-positive proliferating cells could also be detected at the crypt base, intercalating among Paneth cells. Crypt base stem cell proliferation was also increased by *Slc7a5*^ΔIEC^ (Fig. 2C). On the other hand, *in situ* hybridization with probes for stem cell markers LGR5 and OLFM4 mRNAs showed no difference between *Slc7a5*^ΔIEC^ and *Slc7a5*^fl/fl^ mice (Supplementary Fig. 1).

Since intestinal gross morphology did not change very significantly, the increased cell proliferation in *Slc7a5*^ΔIEC^ mice suggest faster cell turn-over in the epithelium in order to maintain homeostasis. To test this, we carried out kinetic studies on cell turn-over by labeling proliferating cells with a single injection of EdU, followed by euthanizing the mice at 2 h, 8 h, 24 h, 48 h and 72 h post injection (Fig. 2D). Analysis of the EdU-labeled cells in the resulting intestinal cross-sections showed that at 2 and 8 h post injection, the EdU-positive cells per crypt in *Slc7a5*^ΔIEC^ mice were about two-folds of those in *Slc7a5*^fl/fl^ mice (Fig. 2E-F). Furthermore, at 8 h, many EdU-positive cells in *Slc7a5*^ΔIEC^ mice but few in *Slc7a5*^fl/fl^ mice had migrated out of crypts and into villi (Fig. 2E), suggesting faster migration and differentiation in knockout cells. By 24 h, most EdU-positive cells in *Slc7a5*^ΔIEC^ mice had migrated to near the top of the villi while most EdU-positive cells in *Slc7a5*^fl/fl^ mice were still near the lower half of the villi (Fig. 2E), indicating again that the *Slc7a5*^ΔIEC^ led to faster cell migration during epithelium turn-over. The EdU-positive cells reached maxima between 8-24 h and subsequently decreased as they turned over, with all removed by 48 h and 72 h in *Slc7a5*^ΔIEC^ and *Slc7a5*^fl/fl^ mice, respectively (Fig. 2E-F), demonstrating that *Slc7a5*^ΔIEC^ had faster turn-over. Consistently, TUNEL-labeling for apoptotic cells showed that apoptotic cells in small intestine were increased dramatically by *Slc7a5*^ΔIEC^ (Fig. 2G-H). Thus, *Slc7a5*^ΔIEC^ caused faster epithelium turn-over by increasing cell proliferation in the crypt with compensatory increases in cell migration and cell death.

### *Slc7a5*^ΔIEC^ mice had fewer Paneth cells and goblet cells in the small intestine

**Figure 3:**
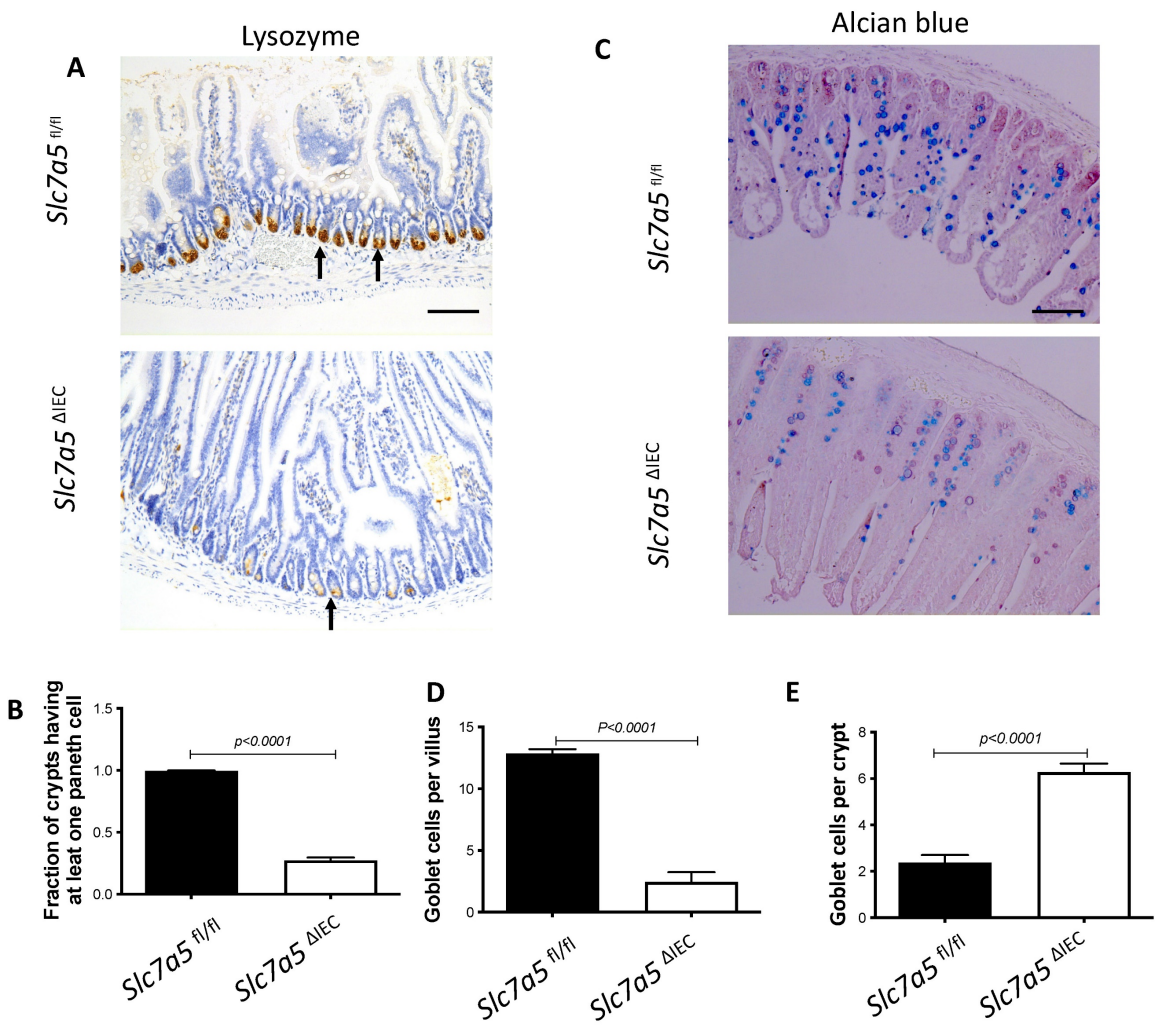
***Slc7a5*^ΔIEC^ causes defects in secretory cell lineages**. **A-B**. Much fewer cells were stained with anti-lysozyme antibody for Paneth cells in small intestine sections of *Slc7a5*^ΔIEC^ mice compared to *Slc7a5*^fl/fl^ mice (A) with quantification of the number of lysozyme-positive Paneth cells/crypt shown in (**B**). Scale bars, 100μm. C-E. Alcian blue stained goblet cells (**C**) were reduced in villi (**D**) but increased in crypts (**E**) of small intestine of *Slc7a5*^ΔIEC^ mice compared to *Slc7a5*^fl/fl^ mice. Scale bars, 100μm. Quantifications were shown as mean ± SD.

We next analyzed if intestinal epithelial cell disruption of *Slc7a5* affected the Paneth cells and goblet cells, the main secretary cells in the small intestine. Immunohistochemical analysis with an antibody against lysozyme, a marker for mature Paneth cells, revealed that *Slc7a5*^ΔIEC^ mice had a dramatic reduction in number of mature Paneth cells compared to *Slc7a5*^fl/fl^ mice (Fig. 3A, B). Similarly, staining with Alcian blue for goblet cells revealed that the number of goblet cells were reduced in villi of *Slc7a5*^ΔIEC^ mice (Fig. 3C, D). Interestingly, more goblet cells were present in the crypts of *Slc7a5*^ΔIEC^ mice (Fig. 3C, E). Thus, *Slc7a5*^ΔIEC^ had abnormal development and/or distribution of the secretory cells***.***

### *Slc7a5*^ΔIEC^ mice have altered intestinal mTORC1 signaling

**Figure 4:**
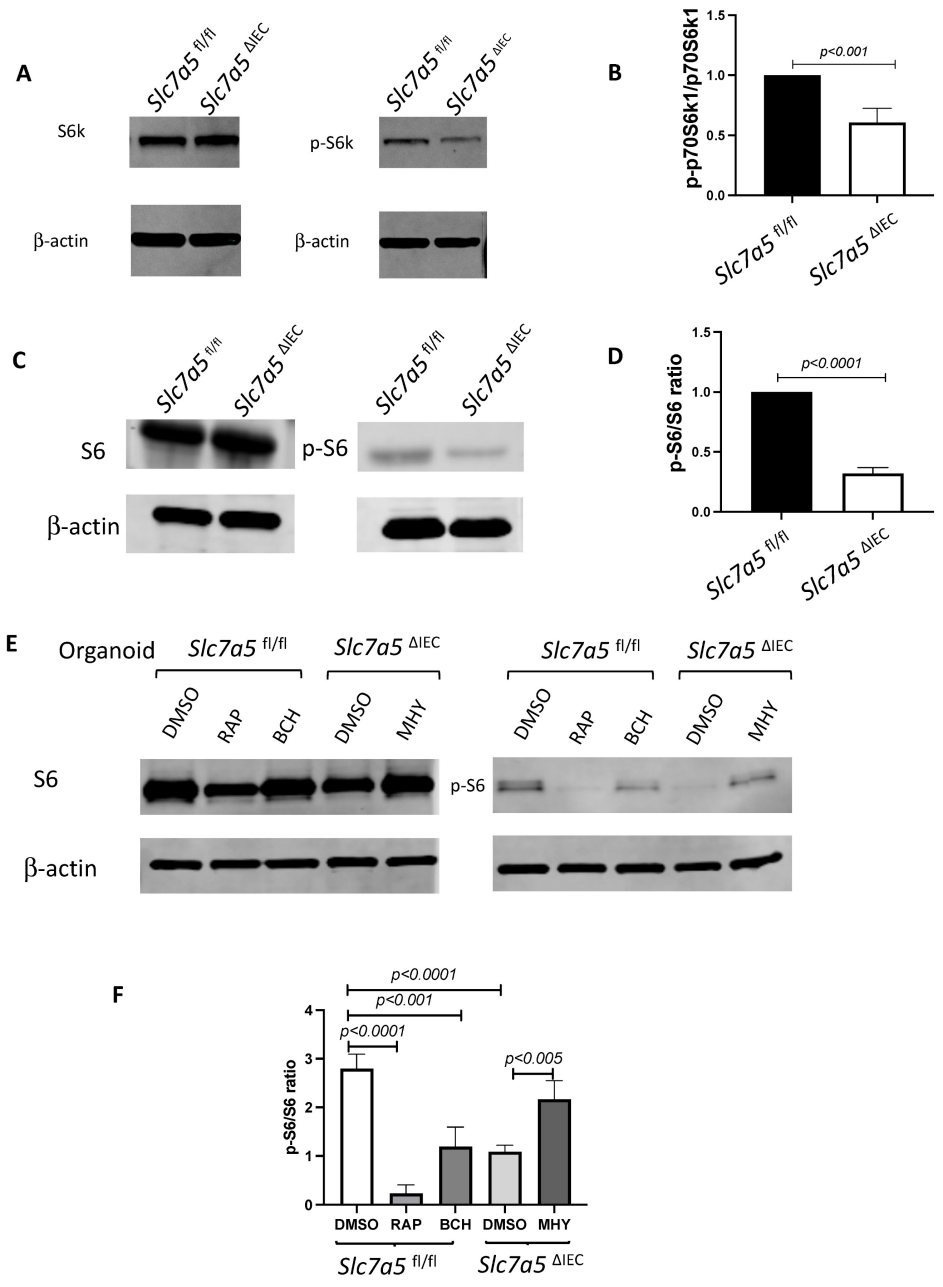
***Slc7a5*^ΔIEC^ leads to reduced mTORC1 signaling in intestinal epithelium**. **A/C**. Western blot analysis of total S6k (**A**) and S6 (**C**), downstream effectors of mTORC1 signaling, and phosphorylated S6k (p-S6k) (**A**) and phosphorylated S6 (p-S6) in intestinal epithelium; β-actin was used as a loading control. B/D. Quantification of the levels of P-S6k (**B**) and P-S6 (**D**), measures of mTORC1 signaling, from 5 (**B**) or 2 (**D**) independent experiments, revealed reduced mTORC1 signaling due to *Slc7a5*^ΔIEC^. **E-F**. *Slc7a5*^ΔIEC^ also reduces mTORC1 signaling in intestinal epithelial organoids *in vitro*. Small intestinal crypts from *Slc7a5*^fl/fl^ and *Slc7a5*^ΔIEC^ mice were cultured as organoids for totally 7 days. Some organoids were treated with BCH (10 mM) starting on day 4 for 3 days while some others were treated with rapamycin (20 μM) or MHY1485 (10 μM) on day 6 for one day, as indicated. On day 7, the organoids were isolated for western blot analysis for indicated proteins (**E**). The signals from the blots were quantified (**F**). Note the reduced p-S6 in *Slc7a5*^ΔIEC^ organoids compared to *Slc7a5*^fl/fl^ mouse organoids. As expected, treatment with MHY1485, an mTORC1 activator, increased p-S6 in *Slc7a5*^ΔIEC^ organoids while both and rapamycin, an mTORC1 inhibitor, and BCH, an SLC7A5 inhibitor, reduced p-S6 in *Slc7a5*^fl/fl^ mouse organoids, resembling *Slc7a5*^ΔIEC^ mice.

*Slc7a5* encodes SLC7A5, also called LAT1, which forms a heterodimeric transmembrane amino acid transporter with glycoprotein CD98 (SLC3A2). The heterodimer can transport L-branched chain amino acids and natural amino acids, leading to activation mTORC1 signaling. In addition, a mTORC1 and mTORC2 knockout study revealed reduced secretory cells 23. To investigate whether *Slc7a5*^ΔIEC^ reduces mTORC1 signaling to affect the secretory cell lineage, we analyzed the phosphorylation of mTORC1 substrates p70 ribosome protein S6 kinase1 (70S6K) and ribosome protein S6 (S6) 24. The results showed that the phosphorylated form of both 70S6K (i.e., p70S6K) (Fig. 4A, B) and S6 (i.e., pS6) (Fig. 4C, D) was reduced in the small intestine of *Slc7a5*^ΔIEC^ mice compared to *Slc7a5*^fl/fl^ mice. To determine if the alteration in mTORC1 signaling was through amino acid transporter activity, we treated intestinal organoids made from intestinal crypts of* Slc7a5*^fl/fl^ and *Slc7a5*^ΔIEC^ mice with mTORC1 activator MHY1485 25, mTORC1 inhibitor rapamycin 25-27, or amino acid transporter inhibitor BCH 11, 28, 29 (Fig. 4E, F). Consistent with the in* vivo* findings, *Slc7a5*^ΔIEC^ organoids had decreased pS6 level compared to *Slc7a5*^fl/fl^ mouse organoids under control condition. Rapamycin treatment of the *Slc7a5*^fl/fl^ mouse organoids led to expected inhibition of pS6. Importantly, MHY1485 treatment of *Slc7a5*^ΔIEC^ organoids increased pS6 to a similar level as in *Slc7a5*^fl/fl^ organoids, while BCH treatment of *Slc7a5*^fl/fl^ organoids reduced pS6 to a level similar to that in *Slc7a5*^ΔIEC^ mouse organoids. These findings not only showed that *Slc7a5*^ΔIEC^ reduced mTORC1 signaling through reduced amino acid transport but also suggest that SLC7A5 is the major amino acid transporter that can regulate mTORC1 signaling in the intestine. On the other hand, we failed to observe reliable cell proliferation or Paneth cell phenotype due to the knockout in the organoids. Since the organoids lacked connective tissue and muscles, the results suggests that cell-cell and cell-ECM (extracellular matrix) interactions are important to reproduce the *in vivo* effects of the knockout, even though cell-autonomous effect on mTOC1 signal could be reproduced *in vitro*.

### *Slc7a5*^ΔIEC^ mice had Paneth dedifferentiation with reduced secretory granules

**Figure 5:**
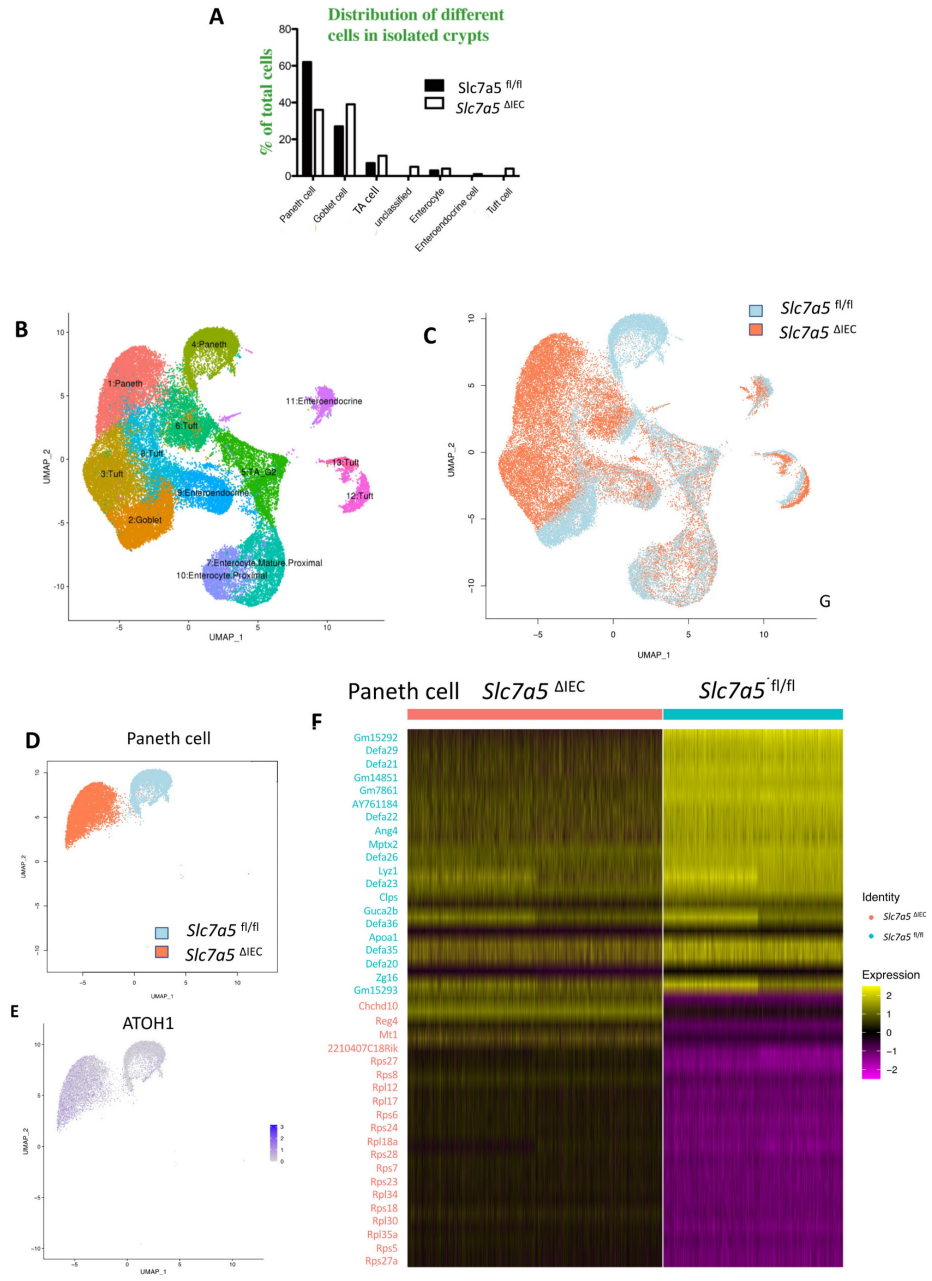
** ScRNA-seq analysis of isolated small intestinal crypts reveals that *Slc7a5*^ΔIEC^ leads to expression of stem cell feature genes in Paneth cells. A**. Percent distribution of small intestinal crypt cells among different cell types after from scRNA-seq. Epithelial cells from *Slc7a5*^fl/fl^ and *Slc7a5*^ΔIEC^ mice were clustered based on t-SNE plot of single cell RNA-seq data as shown Supplementary Fig. 2 and quantified as % of total cells sequenced for each animal type. **B**. Uniform manifold approximation and projection map (UMAP) showing different intestinal epithelial cell types (clusters) in different colors. All cells with scRNA-seq data from both *Slc7a5*^fl/fl^ and *Slc7a5*^ΔIEC^ mice were combined together and subjected to the analysis to show the distribution and location of different types of intestinal epithelial cells in different colors**. C**. The same as in B except the use of blue and orange colors for cells from *Slc7a5*^fl/fl^ and *Slc7a5*^ΔIEC^ mice, respectively. Note that most cells from the *Slc7a5*^fl/fl^ mice and *Slc7a5*^ΔIEC^ mice colocalized on the map. **D**. The view of the regions of the UMAP for Paneth cells from *Slc7a5*^fl/fl^ mice (blue dots) and *Slc7a5*^ΔIEC^ (orange dots) mice. Note that the distinct locations of the Paneth cells from *Slc7a5*^fl/fl^ mice and *Slc7a5*^ΔIEC^ mice suggests significant changes in gene expression patterns between them despite both having Paneth cell gene expression signatures. Similar views for other individual cell types (clusters) from *Slc7a5*^fl/fl^ mice and *Slc7a5*^ΔIEC^ mice are shown as Supplementary Fig. 3. **E**. The expression level of ATOH1 in Paneth cells as obtained from scRNA-seq was mapped on to the UMAP, showing higher levels in *Slc7a5*^ΔIEC^ mice than that in *Slc7a5*^fl/fl^ mice. F. Heatmap showing the relative expression levels of indicated genes in individual Paneth cells from *Slc7a5*^fl/fl^ and *Slc7a5*^ΔIEC^ mice. The genes shown here were features genes distinct for the Paneth cells from *Slc7a5*^fl/fl^ mice and *Slc7a5*^ΔIEC^ mice, with the top 20 for *Slc7a5*^fl/fl^ and bottom 20 for *Slc7a5*^ΔIEC^ Paneth cells.

To further characterize the changes among different types of crypt cells caused by *Slc7a5*^ΔIEC^, we transcriptionally profiled single cells (single cell RNA-seq or scRNA-seq) from the crypts from the small intestine of *Slc7a5*^fl/fl^ and *Slc7a5*^ΔIEC^ mice. After unsupervised graph clustering, the sequenced cells were partitioned into different groups and the cell type identities of the groups were identified based on the expression of known marker genes 30 (Supplementary Fig. 2 and Supplementary Data 1). We found 6 known cell types in the *Slc7a5*^ΔIEC^ crypts and 4 in the *Slc7a5*^fl/fl^ crypts, and another group of unknown identity in the *Slc7a5*^ΔIEC^ sample (Fig. 5A and Supplementary Fig. 2). The two known cell types (enteroendocrine and Tuft cells) not found in the *Slc7a5*^fl/fl^ mouse crypts were likely due to fewer cells were sequenced in the *Slc7a5*^fl/fl^ sample compared to the *Slc7a5*^ΔIEC^ sample since they represented only a small fraction of the total cells (Fig. 5A). Quantification of the percentage of each cell type within the sample for each animal group showed that *Slc7a5*^ΔIEC^ led to increased goblet cell, increased proliferating cells (TA or cell cycle group), and reduced Paneth cells (Fig. 5A), all in agreement with the histology data above. In addition, the feature genes for the group of cells labeled as unclassified cells (the unknown group) were mostly mitochondria genes, which indicated that this group of cells were apoptotic cells, consistent with increased apoptosis in *Slc7a5*^ΔIEC^ intestine as shown above.

To further analyze the scRNA-seq data, we combined all sequenced single cells from *Slc7a5*^fl/fl^ and *Slc7a5*^ΔIEC^ crypts and subjected them to unsupervised graph clustering (UMAP) with different epithelial cell types in different colors (Fig. 5B) or cells from *Slc7a5*^fl/fl^ and *Slc7a5*^ΔIEC^ mouse crypts in different colors (Fig. 5C). Comparison of Fig. 5B and 5C showed that most cell types from *Slc7a5*^fl/fl^ and *Slc7a5*^ΔIEC^ mouse crypts were co-localized. Indeed, when the locations of the cells of individual cell types on the UMAP were analyzed, we found that the cells from *Slc7a5*^fl/fl^ mice and *Slc7a5*^ΔIEC^ mice for most epithelial cell types were largely co-localized (Supplementary Fig. 3) with a small shift for the goblet cells and Tuft cells between the *Slc7a5*^fl/fl^ and *Slc7a5*^ΔIEC^ mice (Supplementary Fig. 3D, F). However, the locations of the Paneth cells from *Slc7a5*^fl/fl^ mice were distinct from the Paneth cells from *Slc7a5*^ΔIEC^ mice (Fig. 5D). When we compared the gene expression profiles of Paneth cells in *Slc7a5*^fl/fl^ and *Slc7a5*^ΔIEC^, we found 35 feature genes upregulated in *Slc7a5*^fl/fl^ Paneth cells compared to *Slc7a5*^ΔIEC^ Paneth cells and 843 feature genes upregulated in *Slc7a5*^ΔIEC^ Paneth cells compared to *Slc7a5*^fl/fl^ Paneth cells (Supplementary Data 1, 2), with the top 20 genes for each group visualized as a heatmap in Fig. 5F. Interestingly, among the top 20 genes expressed highly in *Slc7a5*^fl/fl^ mice were secretory granule genes, such as the *Defa* gene family, *Lyz* gene, and the *Gm* family gene, while those highly expressed in *Slc7a5*^ΔIEC^ mice contained many ribosomal genes, which are associated with regeneration and increased cell stemness 31-34. Finally, GO (Gene Ontology) analysis also showed that GO terms related to anti-microbial and immune responses were most significantly enriched among the genes expressed at higher levels in the *Slc7a5*^fl/fl^ Paneth cells than those in *Slc7a5*^ΔIEC^ Paneth cells, while GO terms related to metabolism and biosynthetic processes were most significantly enriched among the genes expressed at higher levels in the *Slc7a5*^ΔIEC^ Paneth cells than those in *Slc7a5*^fl/fl^ Paneth cells (Supplementary Fig 5 and Supplementary Data 3, 4). These again support the view that loss of SLC7A5 in intestinal epithelial cells causes dedifferentiation of the Paneth cells to gain stem cell features.

To test if the Paneth cells in the knockout mice indeed acquired or had increased stem cell features, we manually matched the 843 feature genes upregulated in *Slc7a5*^ΔIEC^ mice with Harber's stem cell marker genes 30 and identified 5 genes shared between the two (Supplementary Fig 4). In addition, we analyzed all published stem cell marker genes known to facilitate regeneration of the intestinal epithelium 35-42, and found that ATOH1 was upregulated in *Slc7a5*^ΔIEC^ Paneth cells compared to the *Slc7a5*^fl/fl^ cells (Fig. 5E). Since ATOH1+ cells in crypts base are thought to be reserve stem cell, this observation also suggests that Paneth cells gained stem cell features, or had increased stemness, or de-differentiated in the *Slc7a5*^ΔIEC^ mice.

**Figure 6:**
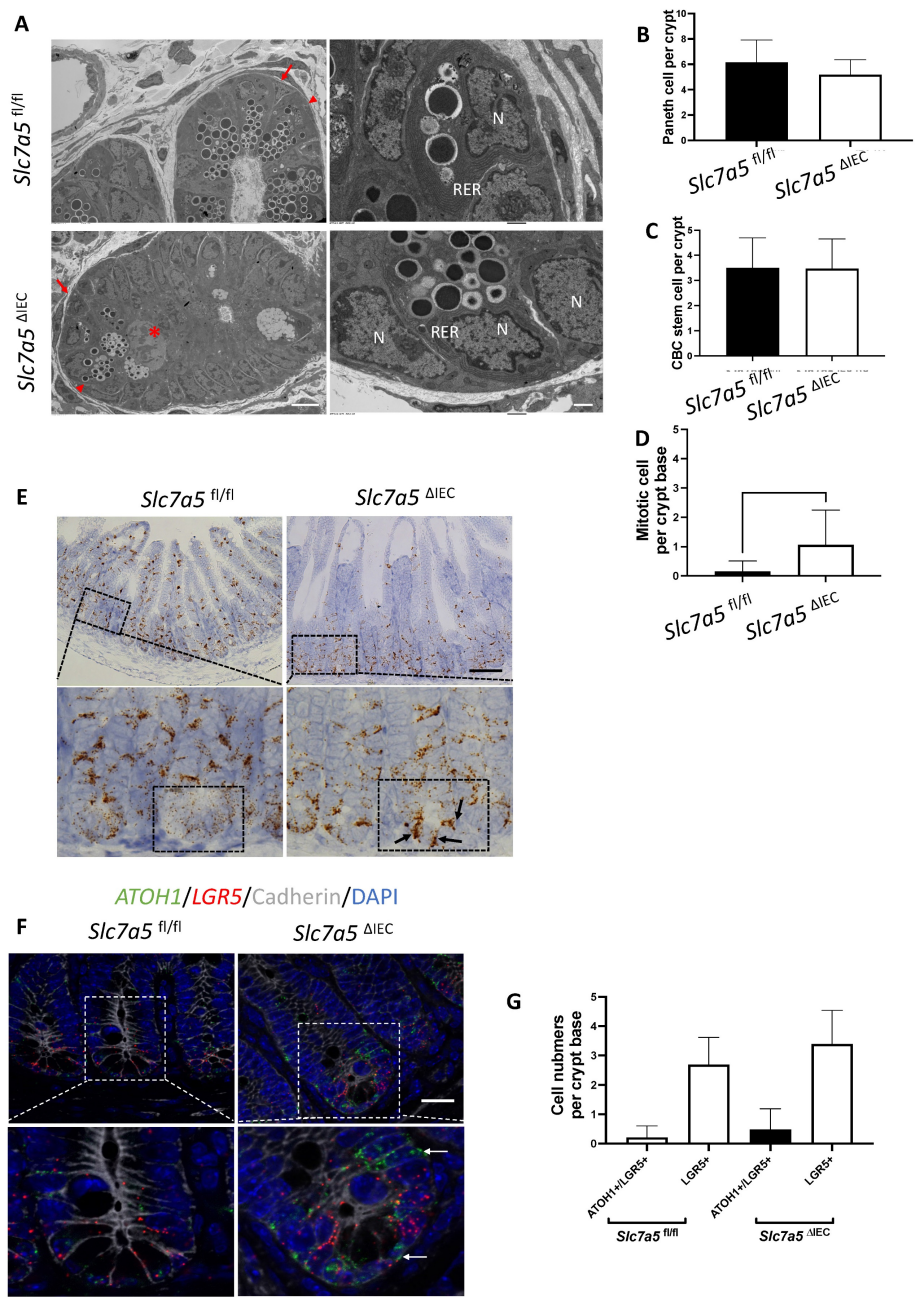
***Slc7a5*^ΔIEC^ secretory granules in Paneth cells and increases crypt base stem cell mitotic activity. A**. Electronic microscopic images of Paneth cells in the small intestine of *Slc7a5*^fl/fl^ and *Slc7a5*^ΔIEC^ mice. Left panel: Scale bars, 6μm. Note that crypt base in *Slc7a5*^ΔIEC^ mice was disorganized, had Paneth cells (red arrowhead) with reduced secretory granules, more mitotic cells (red asterisk). Right panel: Scale bars, 1μm. Note that Paneth cells in *Slc7a5*^ΔIEC^ mice had reduced rough endoplasmic reticulum (right panel). N, nuclei; RER, rough endoplasmic reticulum. Red arrow, stem cells. **B**. Paneth cell numbers were similar between *Slc7a5*^fl/fl^ and *Slc7a5*^ΔIEC^ mice as quantified based on nucleus size and the present of secretory granules in Paneth cells on EM images. **C**. Crypt base stem cell numbers were similar between *Slc7a5*^fl/fl^ and *Slc7a5*^ΔIEC^ mice as quantified based on nucleus size and RER structure of cells on EM images. **D**. Crypt base mitotic cells increased in *Slc7a5*^ΔIEC^ mice as quantified on EM images. **E**. ATOH1 expression was higher in the crypt base of *Slc7a5*^ΔIEC^ mice compared to *Slc7a5*^fl/fl^ mice as detected by single molecule in situ hybridization. The region with a dashed box in each lower panel represents a crypt base, showing increased ATOH1 expression in the *Slc7a5*^ΔIEC^ mice. Scale bars, 100μm. **F**. Co-expression of ATOH1 and LGR5 in some crypt base stem cells in *Slc7a5*^fl/fl^ and *Slc7a5*^ΔIEC^ mice. Single molecule in situ hybridization were performed with a mixture of probes for ATOH1 (green), LGR5 (red), and cadherin (gray) on intestinal cross-sections and the DNA was stained blue. The boxed regions in the top panel were enlarged in the bottom panel. Scale bars, 50μm. White arrow, ATOH1+ cells. **G**. LGR5 and ATOH1 were not co-expressed in most cells. A cell was considered to be positive for an mRNA if at least 5 dots (single molecular hybridization signal) for the mRNA was present in a cell. Results were shown as mean ± SD.

While scRNA-seq data showed a reduction in Paneth cells in *Slc7a5*^ΔIEC^ mice, the reduction was much less than what was observed from lysozyme-staining (Fig. 3A, B). This and the fact that Paneth cells in *Slc7a5*^ΔIEC^ mice appeared to have more stem cell features or be at least partially dedifferentiated prompted us to examine Paneth cells by electron microscopy. Under an electron microscope, the cells at the bottom of the crypts in *Slc7a5*^ΔIEC^ mice were not well organized and the number of secretory granules in Paneth cells were significantly decreased in comparison to *Slc7a5*^fl/fl^ mice (Fig. 6A). In addition, the rough endoplasmic reticulum (RER), where protein synthesis takes place, was also reduced in *Slc7a5*^ΔIEC^ Paneth cells (Fig. 6A), consistent with partial dedifferentiation of Paneth cells. Quantifications based on the ratio of nuclei to cytoplasm and the presence of granules in Paneth cells showed that the number of Paneth cells were similar between *Slc7a5*^fl/fl^ and *Slc7a5*^ΔIEC^ mice (Fig. 6B). These findings thus explained the apparent difference in the changes in Paneth cell number between scRNA-seq and lysozyme-staining since latter detects the presence of lysozyme in the granules. In addition, the electron microscopic analysis also showed that the crypt base stem cell numbers were similar between *Slc7a5*^ΔIEC^ mice and *Slc7a5*^fl/fl^ mice while mitotic cells at the crypt base were increased in *Slc7a5*^ΔIEC^ mice compared to *Slc7a5*^fl/fl^ mice (Fig. 6C, D), consistent with histological analyses above (Fig. 2C, Supplementary Fig. 1).

To further investigate the potential dedifferentiation of the Paneth cells in *Slc7a5*^ΔIEC^ mice, we used single molecule *in situ* hybridization (smISH) and immunofluorescence to detect ATOH1, LGR5, Ki67 and lysozyme at crypt base. The smISH analysis revealed that ATOH1, whose high-level expression has been implicated to a marker for reserved stem cells 36, 37, was highly expressed in the crypt base of *Slc7a5*^ΔIEC^ mice compared to *Slc7a5*^fl/fl^ mice (Fig. 6E). By co-staining for LGR5, a marker for CBC stem cells, or lysozyme, a marker for mature Paneth cells, we found that ATOH1 mRNA was mostly co-expressed with lysozyme (Supplementary Fig. 6A, D) (note that the fraction of ATOH1-positive cells with lysozyme was decreased a little in *Slc7a5*^ΔIEC^ mice, likely due to reduced granules that made some Paneth cells not positive for lysozyme detection in *Slc7a5*^ΔIEC^ mice), but not with LGR5 (Fig. 6F, G). Thus, ATOH1 is largely a marker for Paneth cells at crypt base but its expression was increased in the Paneth cells in *Slc7a5*^ΔIEC^ mice, in agreement with the scRNA-seq data (Fig. 5E). In addition, co-staining for Ki67 showed that in *Slc7a5*^ΔIEC^ mice, there were proliferating cells at the crypt base with ATOH1 expression (Supplementary Fig. 6B, E), likely due to increased proliferation of some stem cells expressing both LGR5 and ATOH1 (Fig. 6F, G). Furthermore, 80% LGR5-positive cells were stained positively for Ki67, compared to 30% in *Slc7a5*^fl/fl^ mice (Supplementary Fig. 3), indicating that *Slc7a5*^ΔIEC^ increased the proliferation of CBC stem cells, consistent with the data from Ki67 staining alone (Fig. 2C).

## Discussion

Amino acids are key inputs of mTORC1 signaling and thus many studies on mTORC1 function in intestinal homeostasis have used calorie restriction as the model. However, results from different studies are sometimes inconsistent. Notably, Yousefi et al. 21 showed that calorie restriction enhanced intestinal epithelial regenerative capacity through a cell-autonomous mechanism by directly downregulating mTORC1 in reserve stem cell under basal conditions but allowing for robust mTORC1 activation in response to radiation injury to a greater extent than in *ad libitum*-fed mice. However, Igarashi et al. 19 and Yilmaz et al. 20 showed that caloric restriction mainly increased CBC stem cell pool through a non-cell-autonomous mechanism by directly downregulating mTORC1 in Paneth cells, which secrete cyclic ADP ribose to activate neighbor LGR5-positive stem cells, causing their expansion. Despite the difference, both studies suggest nutrition shortage can affect different cell types via mTORC1 signaling. In addition, it was reported that disruption of mTORC1 signaling caused a reduction in Paneth cell number and led to less regeneration capacity after radiation injury 23. These various studies suggest that alteration of mTORC1 signaling can influence epithelial cell regeneration.

The present study provides further evidence on the role of mTORC1 signaling in intestinal homeostasis by introducing a genetic model in which mTORC1 signaling is decreased via knockout of the amino acid transporter SLC7A5 in intestinal epithelial cells. We showed that loss of SLC7A5 caused Paneth cell de-differentiation, leading to disruption in secretory function through a reduction in secretory granules. The overall phenotype of *Slc7a5*^ΔIEC^ mice is similar to that of the mTORC1 intestinal epithelial cell-specific knockout mouse 23.

The mTORC1 signaling is generally associated with cell proliferation. As an activator of mTORC1 signaling through amino acid transport, SLC7A5 may be expected to be associated with cell proliferation. For example, cancer cells require a massive nutrient supply and SLC7A5, as an amino acid transporter, has been widely studied in various cancers, highlighting the importance of amino acid transport in cell proliferation and tumor growth 43-45. In addition, in KRAS-mutant colorectal cancer, *Slc7a5* disruption abrogates tumor cell growth 46. Our *Slc7a5*^ΔIEC^ mice expectedly had a reduction in mTORC1 signaling in the intestinal crypts but surprisingly led to increased proliferation of crypt base stem cells and transit amplifying cells. It is puzzling how the decrease of mTORC1 causes an overall increase in cell proliferation. Our scRNA-seq data showed that Paneth cells had increased expression of stem cell feature genes, like ATOH1 and ribosomal genes. Ribosomal genes have been widely linked to carcinogenesis and stem cells 31, 33, 34. Paneth cells have been shown to acquire a stem cell-like transcriptome post-injury and are able to regenerate the whole intestinal epithelium through activation of Notch signaling 47. ATOH1 functions as an essential downstream marker of Notch pathway 48, 49 and ATOH1+ cells can regenerate the whole intestinal epithelium after injury based on a physical lineage tracing study 37. Thus, it is possible that loss of intestinal epithelial cell SLC7A5 causes Paneth cell dedifferentiation that may eventually lead to highly proliferative stem cells and transit amplifying cells, although we did not observe a significant increase in crypt base stem cell number.

Alternatively, the reduction in mature Paneth cells and increase in goblet cells in the crypts could enhance cell proliferation indirectly. It is well known that Paneth cells can function as stem cell niche to influence stem cell identity and proliferation 19, 20, 50, 51. Thus, the dedifferentiation of Paneth cells and/or increased crypt goblet cells due to *Slc7a5*^ΔIEC^ may alter the niche and/or other secreted factors to increase the proliferation of stem cells and transit amplifying cells. It is also possible that increased cell proliferation in *Slc7a5*^ΔIEC^ mice may be secondary to epithelial damage caused by secretary cell dysfunction. Such loss of protection from AMPs and other external factors 8-10, 52 may cause intestinal inflammatory stress, which in turn leads to increased epithelial cell death. The resulting loss of epithelial cells can then generate a compensatory increase in cell proliferation to maintain intestinal homeostasis (Graphic abstract) 53-55. Clearly, further studies are needed to decipher how SLC7A5 regulates intestinal homeostasis through mTORC1 signaling. Regardless of the exact mechanism, our findings highlight an important role of amino acid transport and/or nutritional status in regulated Paneth cell function to affect intestinal homeostasis and pathogenesis.

## Materials and Methods

### Animals

*Slc7a5*^fl/fl^ mice were previously described 17. Villin-Cre (The Jackson Laboratory; stock no. 021504) mice, backcrossed to C57BL/6J mice for more than 10 generations, were bred with *Slc7a5*^fl/fl^ mice to produce *Slc7a5*^ΔIEC^ mice (Fig. 1A). *Slc7a5*^fl/fl^ mice were use as the wild type controls for *Slc7a5*^ΔIEC^ mice. The *Slc7a5*^ΔIEC^ and *Slc7a5*^fl/fl^ mice were sex- and age- matched littermates and cohoused. All mice were maintained in accordance with the NIH animal facility guidelines for laboratory animal research. All animal care and treatments were done as approved by Animal Use and Care Committee of *Eunice Kennedy Shriver* National Institute of Child Health and Human Development, National Institutes of Health.

### Measurements for small intestine, intestinal crypts, villi, and body

The body length and body weight were measured on euthanized* Slc7a5*^fl/fl^ and *Slc7a5*^ΔIEC^ mice from the tip of the mouth to the base of the tail. The length of dissected small intestine was measured from the beginning of the duodenum to the end of the ileum. The length of the villus was measured from the mouth of the crypt to the tip of the villus on H&E-stained intestinal cross-sections. For the isolation of intestinal epithelial cells, the intestine was cut into 2-5 mm segments and incubated in phosphate-buffered saline (PBS) containing 20 mM EDTA for 30 min on ice on a rocker. Epithelial cells were released by vigorous shaking or pipetting and passed through a 70 mm strainer to collect enriched crypts or all the filtrate was used to collect intestinal epithelial cells, which were then washed with cold PBS containing 0.1% bovine serum albumin (BSA).

### Mouse intestinal organoids

IntestiCult Organoid Growth Medium (mouse) (Stem Cell Technologies) was used to establish and culture mouse intestinal organoids. Briefly, the mice were killed and the whole small intestine was harvested from each mouse. The intestine was flushed gently with cold PBS. Scissors were used to make a longitudinal incision along the entire length of the intestine, and the intestine was then cut into 2-5 mm pieces. A 20-ml serological pipette was used to wash the intestinal pieces by pipetting up and down three times. The intestinal pieces were sedimented by centrifugation and re-suspended into fresh PBS. This wash procedure was repeated 15-20 times. Then, the intestinal pieces were suspended in 20 mM EDTA for 30 min on ice on a rocker before pelleting by centrifugation. They were then resuspended in 20 ml of cold PBS containing 0.1% BSA and pipetted up and down three times. The supernatant was then passed through a 70-mm strainer to collect the enriched crypts in the filtrate. This procedure was repeated 3-6 times to obtain enough crypts for organoid culture. The quality of the crypts in the suspension was assessed, and the number of crypts were counted with an inverted microscope. Crypts were then cultured in domes made with a mixture of Matrigel (Corning) and IntestiCult Organoid Growth Medium (1:1). Fifty microliter of the mixture was pipetted into a 24-well plate. Next, the plate was incubated at 37 °C for 10 min to allow the Matrigel to solidify. Finally, 500μl complete IntestiCult Organoid Growth Medium were added to each well and the plate was then incubated at 37 ℃ and 5% CO_2_ for 7 days with the medium changed every other day. For immunoblotting assays, 10 mM BCH were incubated for three days before harvest; 20 μM rapamycin, or 10 μM MHY1485 for overnight.

### EdU staining

Proliferating cells were labeled with 5-ethynyl-2'-deoxyuridine (EdU)-incorporation. Click-iT^™^ EdU Cell Proliferation Kit for Imaging (Thermo Fisher Scientific, Alexa Fluor^™^ 594 dye) was applied following manufacturer's protocol. Briefly, for cell proliferation assay, mice were intraperitoneally injected with EdU (1 mg per mouse) two hours before killing**.** In EdU pulse-chase assay, mice were intraperitoneally injected with EdU (1 mg per mouse), and the mice were killed at 2 h, 8 h, 24 h, 48 h or 72 h, after injection. The EdU-labeled intestine was isolated for paraffin sections (5 µm) as described above. The intestinal paraffin sections were baked at 60 °C for 1 h followed by deparaffinized with xylene and rehydrated through a graded series of ethanol. Then the sections were incubated with Click-iT^®^ Plus reaction cocktail for 30 minutes at room temperature, washed once with 1 × PBST (1 × PBS and 0.05% Tween-20), mounted on glass slides with ProLong^®^ Gold Antifade Mountant with DAPI (Thermo Fisher Scientific). The fluorescent pictures for different colors and different sections were taken under the same settings and then analyzed by using ImageJ at the same setting to count the positive cells.

### Immunohistochemistry

The intestine was removed from age-matched *Slc7a5*^fl/fl^ and *Slc7a5*^ΔIEC^ littermates, flushed with ice-cold 1xPBS buffer, and fixed in 4% formaldehyde (and if needed, stored at 4 °C), followed by embedding in paraffin and then cutting to 5 µm sections. For H&E staining, the 5 µm sections were stained with hematoxylin and eosin following manufacturer's protocol (Sigma) and analyzed under a bright field microscope. For immunofluorescent staining, four to five 5 μm paraffin sections/animal were deparaffinized in xylene and rehydrated in a series of different concentrations of ethanol. Antigen retrieval was performed by boiling in an antigen retrieval buffer (1 mM Tris, 1 mM EDTA and 0.05% Tween-20) for 3 min at 125 °C followed by washing the slides under running water and rinsing them in 1xTBS-Tween (Tris buffered saline plus 0.1% tween-20) for 5 min. After incubation in blocking buffer (10% normal goat serum in PBS) for 1 h at room temperature, the primary antibody was added, and the slides were incubated at 4 °C overnight. The slides were then washed in 1xTBS-Tween and subsequently incubated with a fluorescence-labeled secondary antibody for 1 h at room temperature, before the slides were washed three times with 1x TBS-Tween and covered with DAPI-containing mounting medium to counterstain the DNA. The fluorescent pictures for different colors and/different sections were taken under the same settings. The fluorescent pictures were analyzed by using the Image J at the same setting to count positive cell numbers. The primary antibodies used were anti-lysozyme antibody (Abcam, and Dako) and Ki67 (Cell Signaling Technology). For immunohistochemical staining, paraffin sections of the intestine were incubated with the primary antibody for 1 h at room temperature, followed by washing steps with PBS as described above for immunohistochemistry. The HRP/DAB (ABC) Detection kit (Abcam) was used to detect the signals by following the manufacturer's protocol. The brown Paneth cells in the crypt were then visually counted double-blindly.

### Apoptosis detection

Apoptosis was analyzed with terminal deoxynuclotidyltransferase-mediated dUTP nick-end labeling (TUNEL; Roche) following the manufacturer's protocol. Briefly, the TUNEL *in situ* Cell Death Detection Kit was used on 5 μm paraffin sections of the intestine. Sections were dewaxed with xylene and rehydrated with a series of different concentrations of ethanol. Antigen retrieval was performed by microwaving the sections (700W; 1.5 min) in sodium citrate buffer (pH 6.0) followed by rinsing in PBS. The sections were incubated for 30 min in 0.1 M Tris-HCL pH 7.5, containing 1.5% BSA, and 20% normal bovine serum, washed in PBS, and incubated with TUNEL reaction mixture at 37 °C for 1 h. After removing the TUNEL reaction mixture, sections were washed in PBS several times and counterstained with DAPI. The fluorescent pictures for different colors and/different sections were taken under the same settings and then analyzed by using Image J at the same setting to count the positive cell numbers.

### Alcian blue staining for goblet cells

Paraffin sections of the intestine were stained with Alcian Blue Kit (Abcam) following the manufacturer's protocol. The blue goblet cells in the crypt and villus were counted visually or analyzed by using Fiji Image J.

### *In situ* hybridization

*In situ* hybridization with RNAscope 2.5 HD Reagent Kit-Brown (322371; Advanced Cell Diagnostics, Newark, CA) or RNAscope Multiplex Fluorescent V2 Assay (323100; Advanced Cell Diagnostics, Newark, CA) was performed on 5 μm, formalin-fixed, paraffin-embedded sections according to manufacturer's instructions. The RNAscope probes used were LGR5, OLFM4, ATOH1, the negative control probe *DapB*, and the positive control probe *Ppib*. The positive signals in crypt were quantified by using Fiji Image J or Imaris.

### Western blot

Total proteins from intestinal epithelial cells or intestinal organoids were extracted with M-PER Mammalian Protein Extraction Reagent (Thermo Fisher Scientific) with proteinase inhibitor (Roche) and phosphatase inhibitor (Thermo Fisher Scientific) and then subjected to SDS-polyacrylamide gel electrophoresis, followed by transferring to polyvinylidene difluoride membranes and subsequent immunoblotting assay. Quantification was performed with Li-COR analysis system (LI-COR Bioscience). The antibodies used were anti-phospho-p70S6K1 (Cell Signaling Technology), anti-p70S6K1 (Cell Signaling Technology), anti-phospho-S6 (Cell Signaling Technology), anti-S6 (Cell Signaling Technology), anti-β-actin (Abcam).

### scRNA-seq and data analysis

The enriched intestinal crypts as isolated above were washed twice in PBS, centrifuged at 300 g for 3 min, and the epithelial cells dissociated with TrypLE express (Invitrogen) for 1 min at 37 °C. The dissociated single cells were stained with an EpCAM (Thermo Fisher Scientific), CD45 (Thermo Fisher Scientific), and propidium iodide (Thermo Fisher Scientific). The EpCAM+ CD45- live cells were sorted by flow cytometry and cells was counted. The estimated ideal number of cells were used for scRNA-seq according to manufacturer's instructions (10x Genomics kit, chromium single cell 3' reagent kits V2). Single cell sequencing was performed with Illumina HiSeq2500 sequencer at Center for Cancer Research Sequencing Facility, National Cancer Institute, National Institutes of Health. For analysis of scRNA-seq data, a total of 25,776 cells, 6559 cells from *Slc7a5*^fl/fl^ mice and 19217 cells from *Slc7a5*^ΔIEC^ mice, after filtering and removing the immune clusters, were used. 10x Genomics Cell Ranger software (version 3.1.0) was used for single cell demultiplexing, alignment, filtering, barcode counting, and UMI counting. Then the counting outputs were processed with Seurat package (v3.2.3) under R (version 4.0.3) following the standard workflow (https://satijalab.org/seurat/articles/pbmc3k_tutorial.html). Cell type identity was assigned to cell clusters based on feature genes of intestinal epithelial cells subtypes 30. To compare intestinal epithelial cell subtypes between *Slc7a5*^ΔIEC^ and *Slc7a5*^fl/fl^ mice, the counting data of both *Slc7a5*^ΔIEC^ and *Slc7a5*^fl/fl^ mouse samples were aggregated as one with 10x Genomics Cell Ranger software and processed with R Seurat package and the same workflow as above. After assigning cell types to clustered cells, differentially-expressed genes between different clusters were identified with FindMarkers function in R Seurat package with setting: only.pos=True, min.pct=0.25, and logfc.threshold=0.25.

### Electronic microscopy

Mice were transcardially perfused fixed with 4% PFA with 2.5% glutaraldehyde, made in PBS buffer, pH 7.4. Small intestines were removed and left to post-fix overnight in the same fixative at 4 °C. All tissue samples were then rinsed in 0.1M sodium cacodylate buffer. The following processing steps were carried out using the variable wattage Pelco BioWave Pro microwave oven (Ted Pella, Inc., Redding, CA.): post-fixed in 1% osmium tetroxide made in 0.1 M sodium cacodylate buffer, rinsed in double distilled water (DDW), 2% (aq.) uranyl acetate enhancement, DDW rinse, ethanol dehydration series up to 100% ethanol and propylene oxide, followed by a Embed-812 resin (Electron Microscopy Sciences, Hatfield, PA.) infiltration series up to 100% resin. The epoxy resin was polymerized for 20 h in an oven set at 60 °C. Ultra-thin sections were cut on a Leica EM-UC7 Ultramicrotome (90 nm). Thin sections were picked up and placed on 200 mesh cooper grids and post-stained with uranyl acetate and lead citrate. Imaging was accomplished using a JEOL-1400 Transmission Electron Microscope operating at 80kV and an AMT BioSprint-29 camera.

### Statistical analysis

Representative images of at least two independent experiments with each having at least three mice for each sample group were shown. All statistical analyses and graphs were performed/generated by using GraphPad Prism version 8.0 (GraphPad Software, La Jolla, CA). Student's t test was used to examine the differences between groups and a P < 0.05 was considered statistically significant. In the analysis of histology, multiple sections from each animal were used and 10 to 45 sections in total were analyzed for each group. All data were expressed as mean ± SD.

## Supplementary Material

Supplementary figures.

Supplementary tables.
