# Supplementary material for: Amino acid transporter SLC7A5 regulates cell proliferation and secretary cell differentiation and distribution in the mouse intestine
Source: Int J Biol Sci. 2024 Mar 25;20(6):2187–201. doi: 10.7150/ijbs.94297 (PMC11008275; doi:10.7150/ijbs.94297)
Supplement: Supplementary file 1 — Supplementary figures. [file ijbsv20p2187s1.pdf]

Supplementary Figure 1

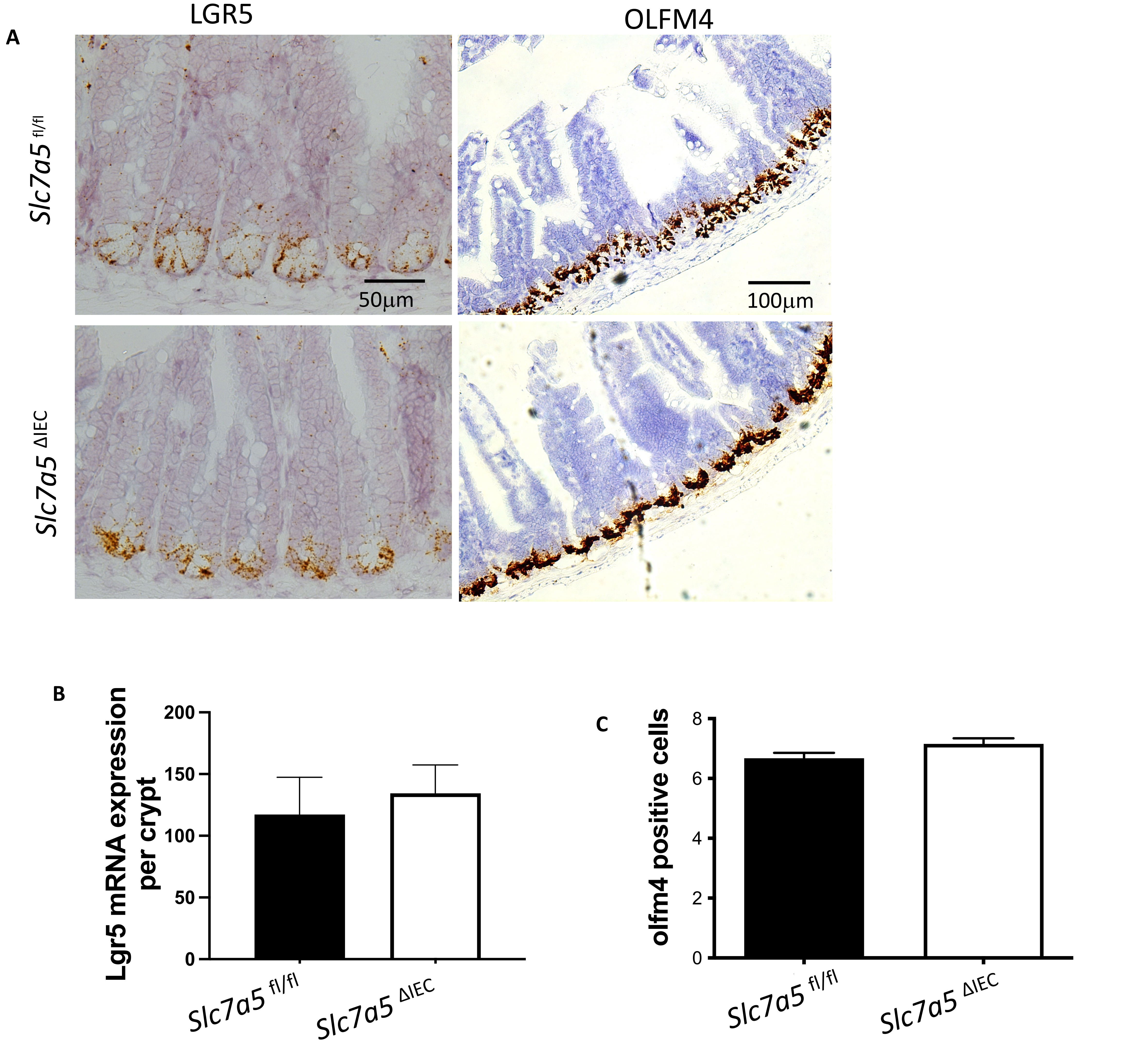

Supplementary Figure 1. *Slc7a5* $^{\Delta IEC}$ does not affect stem cell marker gene expression in the crypt.
A. Representative pictures of LGR5 and OLFM4 single molecule in situ hybridization.
B-C. Quantification of the in situ hybridization data showed that lgr5 mRNA level (**B**) and OLFM4+stem cell numbers (**C**) were similar between *Slc7a5* $^{fl/fl}$ and *Slc7a5* $^{\Delta IEC}$ crypts.

Supplementary Figure 2

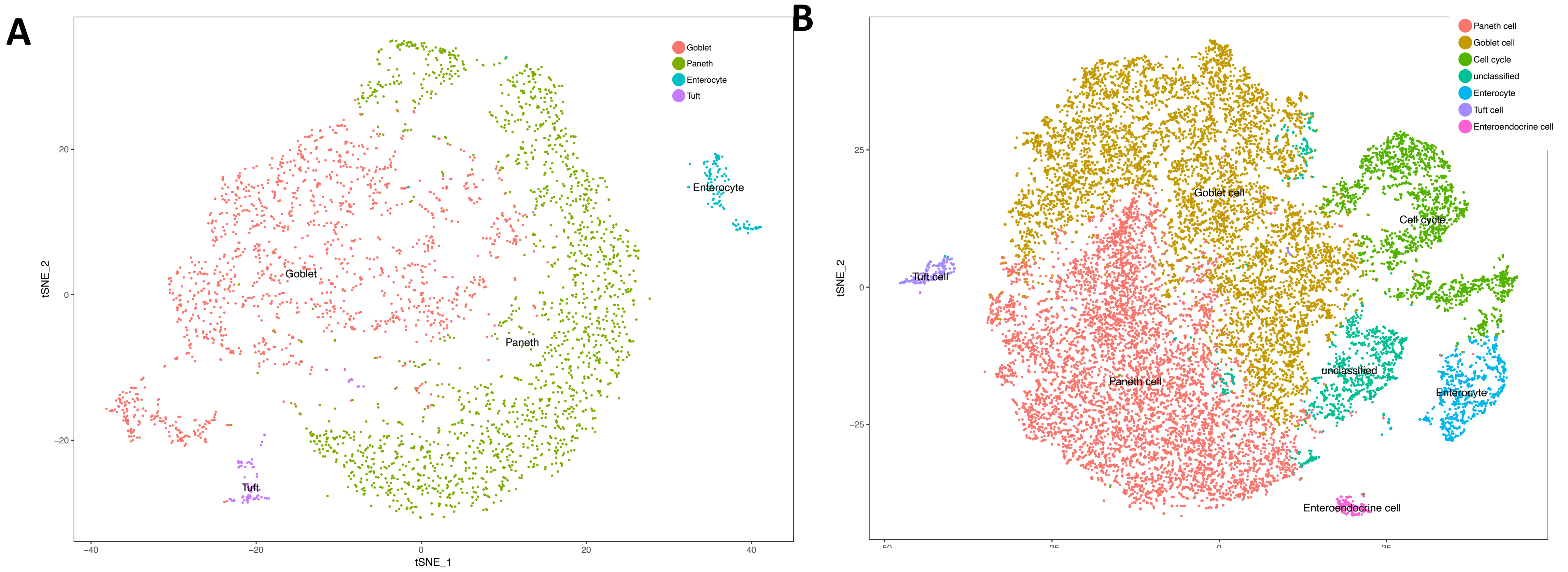

Supplementary Figure 2. ScRNA-seq analysis. Epithelial cells from intestinal crypts of *slc7a5* $^{fl/fl}$ (**A**) and *slc7a5* $^{\Delta IEC}$ (**B**) mice, respectively, were used for scRNA-seq and the cells for each animal type were clustered based on t-SNE plot of the scRNA-seq data with different cell types shown in different colors (note that the cells in cluster labeled as cell cycle were transit amplifying cells or TA cells).

Supplementary Figure 3

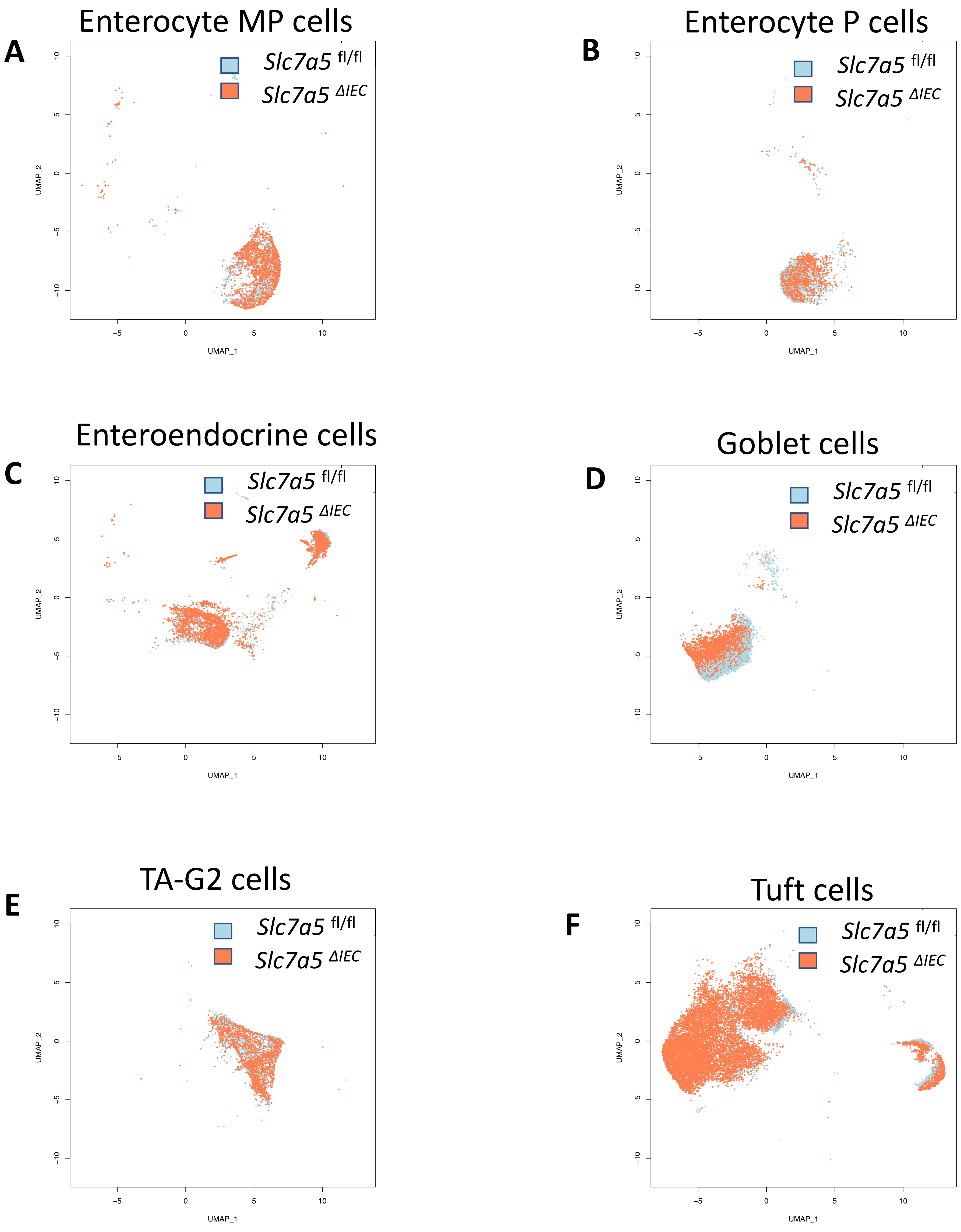

Figure s3. Focused views of the regions of the UMAP (Fig. 5C) for the indicated individual epithelial cell types from *slc7a5* $^{fl/fl}$ mice (blue dots) and *slc7a5* $^{\Delta IEC}$ (orange dots) mice. Note that the co-locations of the cells from the *slc7a5* $^{fl/fl}$ mice and *slc7a5* $^{\Delta IEC}$ mice for these cell types suggest little or few changes in gene expression between *slc7a5* $^{fl/fl}$ mice and *slc7a5* $^{\Delta IEC}$ mice.

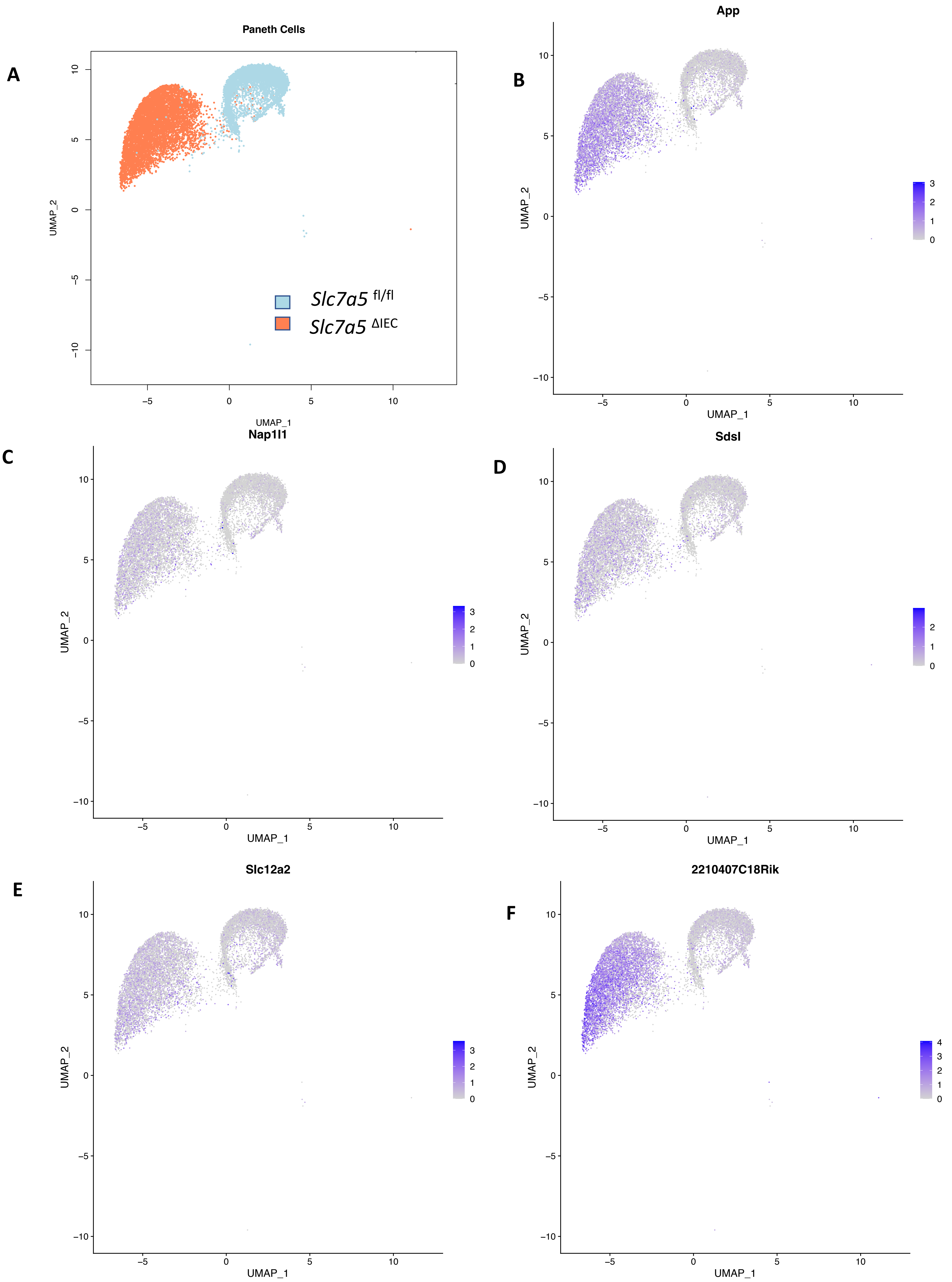

Supplementary Figure 4. UMAP views of increased expression of several stem cell feature genes in the Paneth cells from *Slc7a5* $^{\Delta IEC}$ mice compared to that in *Slc7a5* $^{fl/fl}$ mice.

A. Same image as in figure 5D to show UMAP regions for Paneth cells from *Slc7a5* $^{fl/fl}$ mice (blue dots) and *Slc7a5* $^{IEC\text{-}KO}$ (orange dots) mice.

Supplementary Figure 4

B to F: The expression levels of 5 stem cell feature genes, App, Napl1, Sdsl, slc12a2 and 2210407C18Rik, in Paneth cells as obtained from scRNA-seq were mapped on to the UMAP, showing higher levels in *Slc7a5* $^{\Delta IEC}$ mice than those in *Slc7a5* $^{fl/fl}$ mice.

Supplementary Figure 5

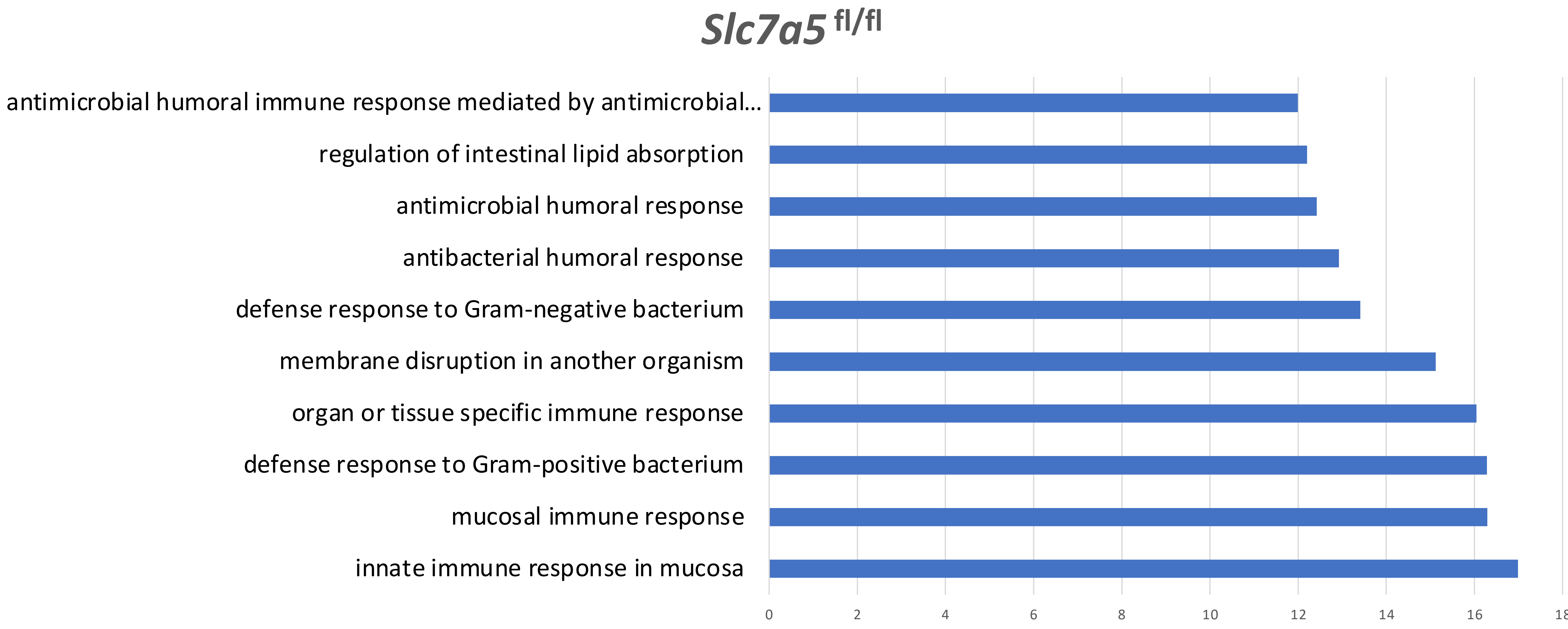

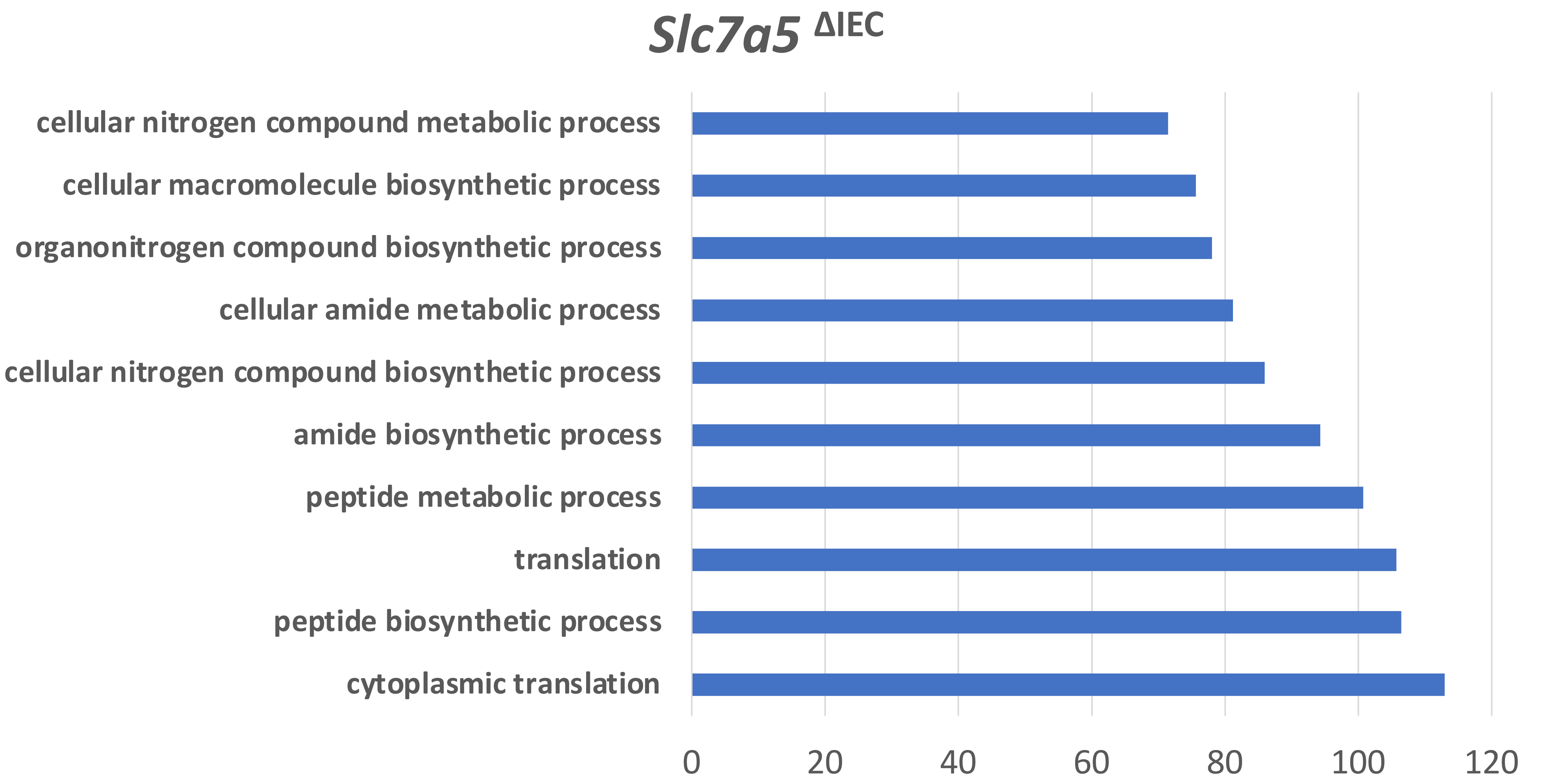

Supplementary Figure 5. GO analysis of DEGs between *Slc7a5* $^{fl/fl}$ mice and *Slc7a5* $^{\Delta IEC}$ mice's Paneth cell.

A. GO terms related to anti-microbial and immune responses were most significantly enriched among the genes expressed at higher levels in the wild type Paneth cells than those in knockout Paneth cells.

B. GO terms related to metabolism and biosynthetic processes were most significantly enriched among the genes expressed at higher levels in the knockout Paneth cells than those in wild type Paneth cells.

Supplementary Figure 6

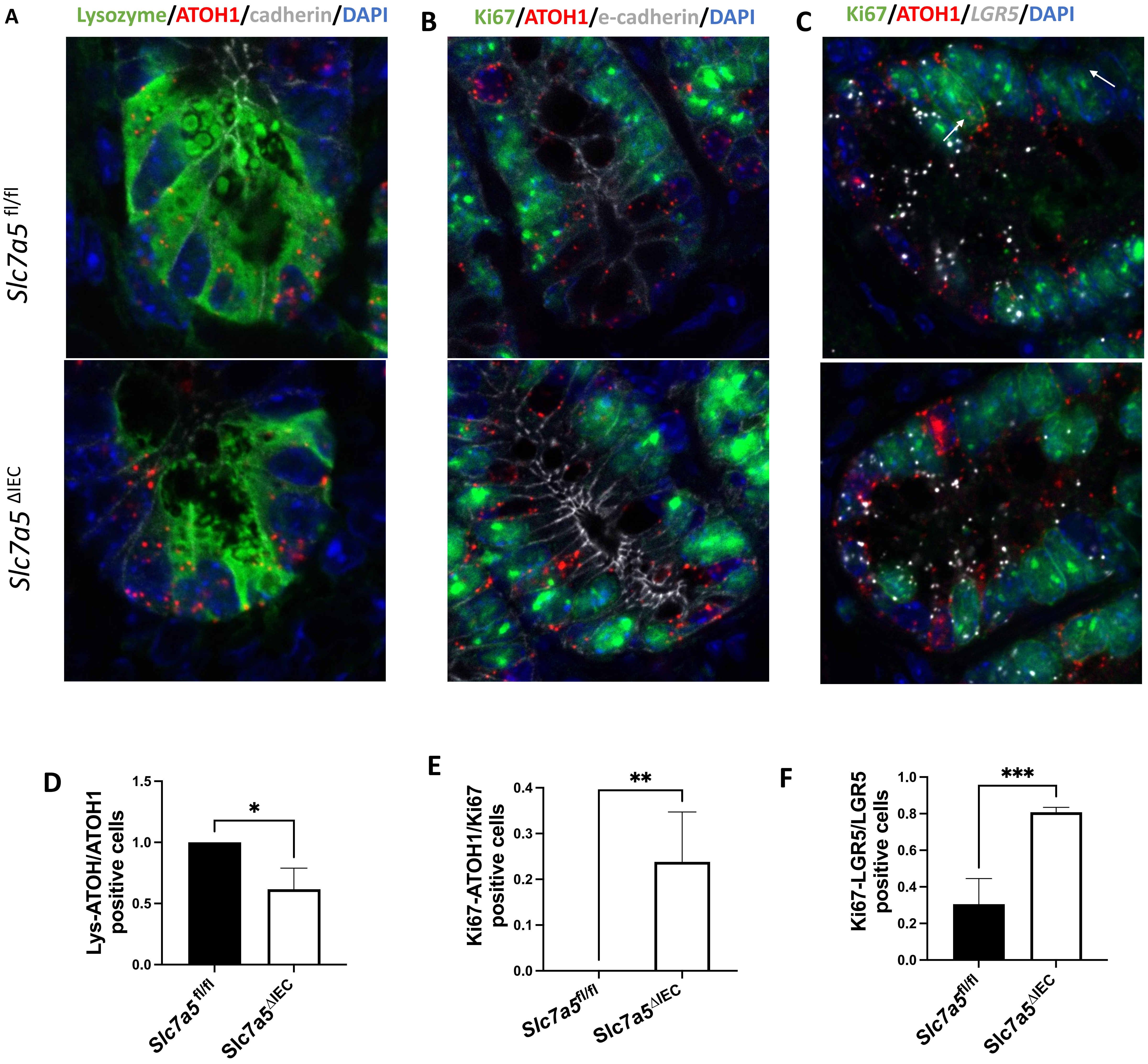

Supplementary Figure 6. Single RNA in situ hybridization and/or immunohistochemical (for lysozyme) analyses of small intestinal crypt base.

A. Representative pictures of lysozyme and atoh1 expression at crypt base in *Slc7a5* $^{fl/fl}$ and *Slc7a5* $^{\Delta IEC}$ mice.

B. Representative pictures of Ki67 and atoh1 expression at crypt base in *Slc7a5* $^{fl/fl}$ and *Slc7a5* $^{\Delta IEC}$ mice.

C. Representative pictures of Ki67, ATOH1, and LGR5 expression at crypt base in *Slc7a5* $^{fl/fl}$ and *Slc7a5* $^{\Delta IEC}$ mice.

D. Quantitative analysis of panel A showing that all ATOH1+ cells were lysozyme-positive in *Slc7a5* $^{fl/fl}$ mice, while only about 60% ATOH1+ cells were lysozyme positive in *Slc7a5* $^{\Delta IEC}$ mice.

E. Quantitative analysis of panel B showing that Atoh1 were expressed in some proliferating cells in the crypt base of *Slc7a5* $^{\Delta IEC}$ but not *Slc7a5* $^{fl/fl}$ mice.

F. Quantitative analysis of panel C showing increased fraction of LGR5+ cells were proliferating in *Slc7a5* $^{\Delta IEC}$ mice crypt base.
